# Metabolic analyses reveal different mechanisms of leaf color change in two purple-leaf tea plant (*Camellia sinensis* L.) cultivars

**DOI:** 10.1038/s41438-017-0010-1

**Published:** 2018-02-07

**Authors:** Jiazhi Shen, Zhongwei Zou, Xuzhou Zhang, Lin Zhou, Yuhua Wang, Wanping Fang, Xujun Zhu

**Affiliations:** 1 College of Horticulture, Nanjing Agricultural University, Nanjing, 210095 China; 2 Department of Plant Science, University of Manitoba, Winnipeg, MB R3T 2N2 Canada; 3 Bureau of Rural Economic Development of Huangdao District, Qingdao, Shangdong 266400 China

## Abstract

Purple-leaf tea plants, as anthocyanin-rich cultivars, are valuable materials for manufacturing teas with unique colors or flavors. In this study, a new purple-leaf cultivar “Zixin” (“ZX”) was examined, and its biochemical variation and mechanism of leaf color change were elucidated. The metabolomes of leaves of “ZX” at completely purple, intermediately purple, and completely green stages were analyzed using ultra-performance liquid chromatography quadrupole time of flight mass spectrometry (UPLC-QTOF-MS). Metabolites in the flavonoid biosynthetic pathway remained at high levels in purple leaves, whereas intermediates of porphyrin and chlorophyll metabolism and carotenoid biosynthesis exhibited high levels in green leaves. In addition, fatty acid metabolism was more active in purple leaves, and steroids maintained higher levels in green leaves. Saponin, alcohol, organic acid, and terpenoid-related metabolites also changed significantly during the leaf color change process. Furthermore, the substance changes between “ZX” and “Zijuan” (a thoroughly studied purple-leaf cultivar) were also compared. The leaf color change in “Zijuan” was mainly caused by a decrease in flavonoids/anthocyanins. However, a decrease in flavonoids/anthocyanins, an enhancement of porphyrin, chlorophyll metabolism, carotenoid biosynthesis, and steroids, and a decrease in fatty acids synergistically caused the leaf color change in “ZX”. These findings will facilitate comprehensive research on the regulatory mechanisms of leaf color change in purple-leaf tea cultivars.

## Plant breeding: Coloring tea leaves

Researchers in China have uncovered how the leaves of purple-leaf tea varieties acquire their color. Wanping Fang and Xujun Zhu of Nanjing Agricultural University analyzed the biochemical profile and gene expression patterns in the young purple leaves and older green leaves of two purple tea varieties, one new and one previously studied. The purple leaves of both varieties had higher levels of red/purple pigments known as flavonoids, as well as increased expression of genes related to their synthesis. However, only the new variety also had increased chlorophyll levels in green leaves. Both varieties are purple because flavonoids mask the color of chlorophyll, but the process is driven by different mechanisms in each. These findings clarify how tea varieties develop different levels of flavonoids, which affect tea quality due to their antioxidant and antimicrobial activity.

## Introduction

Tea plants (*Camellia sinensis* L.) can be used to manufacture non-alcoholic beverages and are extensively cultivated^1^ for different traits and applications. During long-term natural hybridization and under artificial selection pressure, the color of tea leaves has greatly diversified, and this variation has been widely used in recent breeding programs. The albino tea cultivar “AnjiBaicha”, a famous green-revertible tea variety, has white young shoots at low temperatures (< 20 °C), and the albino leaves accumulate higher levels of amino acids than green leaves.^2^ Yellow-leaf tea cultivars have also been developed, one of which exhibits the chlorina phenotype and displays significantly decreased chlorophyll and carotenoid contents but a higher number of amino acids and disrupted chloroplasts (e.g., cultivars “ZH1” and “ZH2”).^3,4^ A light-sensitive cultivar, “Baijiguan”, was identified as another type of yellow-leaf tea—its young leaves show a yellow color under high light intensity but turn green when moved to low light intensity for a certain number of days.^5^ Furthermore, a purple-leaf cultivar, “Zijuan” (“ZJ”), with purple buds, stems, and leaves, has also been cultivated. The purple leaves gradually become green with leaf growth and development.^6^ These tea cultivars are valuable materials for producing unique teas with specific colors and flavors. They are also useful resources as tea germplasm to benefit the whole tea industry in the future.

Flavonoid compounds, especially anthocyanins, are responsible for leaf color and physiological and biochemical processes.^7^ Anthocyanins are secondary metabolites that protect plants against various biotic and abiotic stresses, including cold/freezing,^8^ salt,^9^ and low phosphate stresses.^10^ The anthocyanins in purple tea leaves have various health-related biological functions, such as acting as antioxidants and antimicrobial agents,^11,12^ lowering blood lipids,^13^ and preventing colorectal cancer.^14^ Therefore, the purple color has been employed as a quality parameter in tea breeding programs. Increasing numbers of tea clones with purple leaves, such as cultivars “Wuyiqizhong18”,^7^ “Sunrouge”,^15^ and “Ziyan”^16^ have been developed and reported.

The purple-leaf tea cultivars have been studied for both the chemical composition of tissues and molecular mechanisms of color formation. A number of studies have characterized anthocyanin compositions and quantified anthocyanin contents in purple-leaf tea cultivars.^15,17,18^ The catechin profiles, total polyphenols,^16,19–21^ and antioxidant function of their extracts have also been analyzed.^22^ Research on purple-leaf tea cultivars has made great progress at the molecular level. Using different biotechnologies, researchers have elucidated that anthocyanin accumulation is mainly controlled by later biosynthetic genes (e.g., gene coding for anthocyanidin synthase) and genes involved in anthocyanin transport (e.g., gene coding for UDP-glucosyl transferase) and is regulated by the MYB–bHLH–WDR complex, which consists of CsAN1 (R2R3–MYB transcription factor), CsGL3 and CsEGL3 (bHLH transcription factor), and CsTTG1 (WD-repeat protein).^6,7,23–26^ These studies investigated the biochemical basis and elucidated the mechanism that controls and regulates anthocyanin production. However, the mechanism underlying the change in tea leaf color from purple to green is largely unknown. Metabolite profiling can provide deeper insight into complex regulatory processes to determine the phenotype directly^27^ and to depict the phenotypic variation in plants, including* Arabidopsis*,^28^ maize,^29^ and rice.^30^ However, few studies have focused on the metabolome of purple-leaf tea plants during the color change process.

To better understand the biological process of leaf color change and the dynamic changes of related metabolites, we investigated the metabolic profiles of completely purple, intermediately purple, and completely green leaves of the cultivar “Zixin” (“ZX”), which has purple tender leaves and stems and green mature leaves. The variations in flavonoids, the intermediates in porphyrin and chlorophyll metabolism and carotenoid biosynthesis, fatty acids, steroids and other metabolites, including saponins, alcohols, organic acids, and terpenoid-related metabolites, were profiled. The metabolite changes in cultivars “ZX” and “ZJ” during the color change process were also compared.

## Materials and methods

### Plant materials

**Fig. 1:**
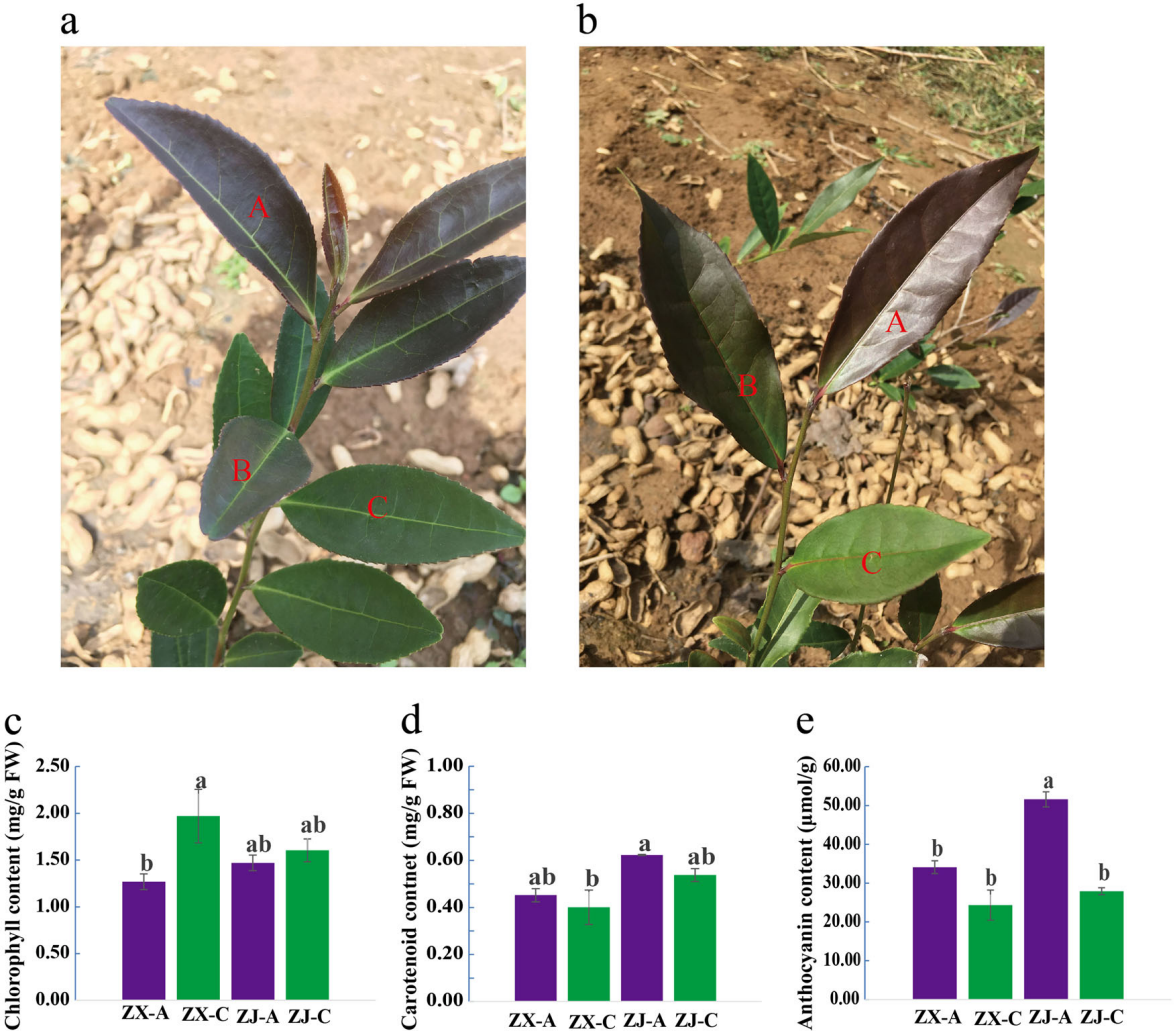
Growth performance and pigment (chlorophyll, carotenoid, and anthocyanin) contents in tea cultivars “ZX” and “ZJ”. Growth performance of (**a**) “ZX” and (**b**) “ZJ” shoots in autumn. The different sampling positions are represented by capital letters. The contents of (**c**) chlorophyll, (**d**) carotenoid, and (**e**) anthocyanin in leaves from the two cultivars are shown as the mean ± S.E.M. Bars with different letters above the columns in the figure indicate significant differences at *P* < 0.05 (Duncan’s multiple range test)

Two-year-old plants of cultivated purple-leaf tea cultivars (*Camellia sinensis* L. cv. ZX) and (*Camellia sinensis* L. cv. Zijuan) were planted in the tea plantation of Huangdao in Shandong Province, China (35°97′ N, 120°18′ E). “ZX” is a new purple-leaf cultivar, which was selected from a natural purple-leaf mutant in Huangdao via systematic selection. The characteristics of the cultivar “ZX” are as follows: the stems, bud, and first two to three leaves are all purple, and the leaf finally turns green, while the leaf vein is evergreen. The leaf of “ZX” has a lustrous surface and is thicker than that of “ZJ”. Healthy, tender shoots were harvested on 30th September, 2016, and were collected separately as completely purple (A), intermediately purple (B), and completely green (C) leaves (Fig. 1a, b). Each sample contained six and four biological replicates for “ZX” and “ZJ” metabolome analysis, respectively. The sampled leaves were immediately frozen in liquid nitrogen andstored at −80 °C for later metabolite and RNA extraction.

### Extraction procedure

Global unbiased metabolite profiling of the samples was performed using ultra-high performance liquid chromatography-tandem mass spectrometry (UPLC–MS/MS). A total of 25 mg of stored leaves was placed in a 1.5-ml tube and aliquoted with 800 μl chilled methanol/ddH_2_O (1:1). Subsequently, two small steel balls were added to each tube. The tubes were then transferred to a TissueLyser LT (QIAGEN, Duesseldorf, Germany) to homogenize the samples at a frequency of 60 HZ for 5 min. The homogenized samples were centrifuged at 25 000 × *g* for 20 min (4 °C).

The supernatant (300 μl) was transferred to a new tube, and 80 μl of supernatant was aliquoted into 96-well plates for subsequent UPLC-QTOF-MS analysis. A “quality control” (QC) sample was established by blending an equal volume (200 μl) of supernatant from each biological sample. The “pooled” sample was used to observe the repeatability within ananalytical batch and to allow removal of metabolic characteristics associated with excessive drift in signal or unacceptable retention time and mass accuracy before data analysis was performed following a quality assurance (QA) process.^31^

### Metabolomic profiling platform

All samples were measured in random order using the ultra-performance liquid chromatography quadrupole time of flight mass spectrometry (UPLC–QTOF–MS) system. All chromatographic separations were carried out using a UPLC system (Waters, Milford, USA). An ACQUITY UPLC BEH C_18_ column (100 × 2.1 mm^2^, 1.7 μm, Waters, Milford, USA) was used for the reversed phase separation. The temperature of the column was maintained at 50 °C. The mobile phase contained solvent A (water with 0.1% formic acid) and solvent B (acetonitrile with 0.1% formic acid). The flow rate of the mobile phase was 0.4 ml min^−1^. Gradient elution procedures were set as follows: 0–2 min, 100% eluent A; 2–11 min, 0–100% eluent B; 11–13 min, 100% eluent B; 13–15 min, 100% eluent A. Extraction solutions (10 μl) of each sample were injected into the UPLC–QTOF–MS system.

The metabolites eluted from the column were detected using a high-resolution tandem mass spectrometer SYNAPT G2 XS QTOF (Waters, Milford, USA). The Q-TOF was operated in both positive and negative ion modes. In the positive ion mode, the capillary and sampling cone voltages were 2 kV and 40 V, respectively, compared with 1 kV and 40 V in the negative ion mode. The mass spectrometry data were collected in centroid MSE mode. The TOF mass range was set from 50 to 1200 Da, and the scan time was 0.2 s. For MS/MS detection, all precursors were fragmented using 20–40 eV voltage and were scanned for 0.2 s. During the acquisition, the LE signal was acquired every 3 s for calibrating the mass accuracy. Furthermore, to evaluate the stability of the LC–MS during the whole acquisition, a QC sample was acquired after every 10 samples.

### Data analysis

Raw data pretreatment, including peak alignment, peak extraction, normalization, deconvolution, and compound identification, was carried out using Progenesis QI software (version 2.2, Waters, Milford, MA, USA). To obtain stable variables, a threshold for the relative standard deviation (RSD) of metabolites in the QC samples was set at 30%, which was regarded as a criterion in the assessment of repeatability in the metabolomics data sets. In this study, the retained peaks were normalized to the QC sample using quality control–based robust LOESS signal correction.^31,32^

The filtered data were loaded into Simca-P software (version 13.0, Umetrics AB, Umea, Sweden) for multivariate statistical analyses, including principal components analysis (PCA) and partial least squares discriminant analysis (PLS-DA). Discriminating compounds were selected based on a statistically significant threshold of variable influence on projection (VIP) values acquired from the PLS-DA model and were then validated using an FDR (false discovery rate) test from the R statistical toolbox (R 3.0.3, www.r-project.org) with *p* < 0.05. Metabolites with VIP ≥ 1.0 and fold change ≥ 1.2 or fold change ≤ 0.8 and q-value ≤ 0.05 were considered as discriminating compounds between two samples.

Hierarchical clustering analysis (HCA) was carried out using R software (version3.0.3). The data were log 2 transformed, and similarity assessment for clustering was based on the Euclidean distance coefficient.

### Metabolite identification

The identification of compounds detected by LC–MS was carried out based on a search of accurate masses of significant peak features against the online KEGG (http://www.kegg.jp/) and HMDB (http://www.hmdb.ca) databases. A metabolite name was reported when the mass difference between observed and theoretical compounds was <10 ppm. Putative identities were further confirmed by LC–MS/MS using QTOF (Waters, Milford, USA). Primary identification was performed by matching the exact mass and the isotopic distribution of the targeted ions. MS/MS fragmentation spectra were analyzed to determine the structure of the fragmented molecules or were compared with the spectral data of available reference compounds in specific databases (HMDB, KEGG).^32^

### Pigment content analysis

The determination of chlorophyll contents was performed, as previously reported^33^ with a minor modification. A total of 50 mg of leaves from all samples wereground on ice with 10 ml 95% ethanol (v/v). Then, the extracts were filtered and brought to a volume of 25 ml using 95% ethanol (v/v). Finally, the chlorophyll extracts were analyzed using a UV5800 ultraviolet spectrophotometer (METASH, Shanghai, China). The absorbance readings were performed at 665 nm, 649 nm and 470 nm, respectively. Total chlorophyll contents were calculated as previously reported.^34^ All analyses were repeated three times.

The total anthocyanin content was measured using a UV5800 ultraviolet spectrophotometer (METASH, Shanghai, China) following the method described by Wei et al. (2016).^24^

### Quantitative real- time PCR analysis

Total RNA was isolated from 100 mg of leaves using a plant RNA extraction kit (Aidlab, China). TransScript® One-Step gDNA Removal and cDNA Synthesis SuperMix (TransGen, China) was used for genomic DNA digestion and first-strand cDNA synthesis. Quantitative real-timePCR (qRT-PCR) was carried out on a Roche lightCycler 480 machine (Basel, Kanton Basel-Stadt, Switzerland). The primer pairs used for qRT-PCR are shown in Supplementary Table 1. Reference genes of *Camellia sinensis* GAPDH were used as an internal control. For each gene, all experiments were repeated three times per sample. Relative gene expressions were calculated using the 2^−ΔΔCt^ method.^35^ The transcripts levels are presented as the mean ± standard error mean (S.E.M.).

## Results

### Metabolomic analysis of LC–MS data

In total, 3813 (88.08%) and 4716 (78.60%) clean retention time-exact mass pairs were obtained in each sample profile by HPLC–MS and subsequent analysis in positive and negative ion modes, respectively. There was no drift in the total ion chromatography plot of the QC samples in either ion mode (Supplementary Figure 1). Hence, the metabolic features demonstrated that LC–MS profiling was acceptably reproducible and stable.

**Fig. 2:**
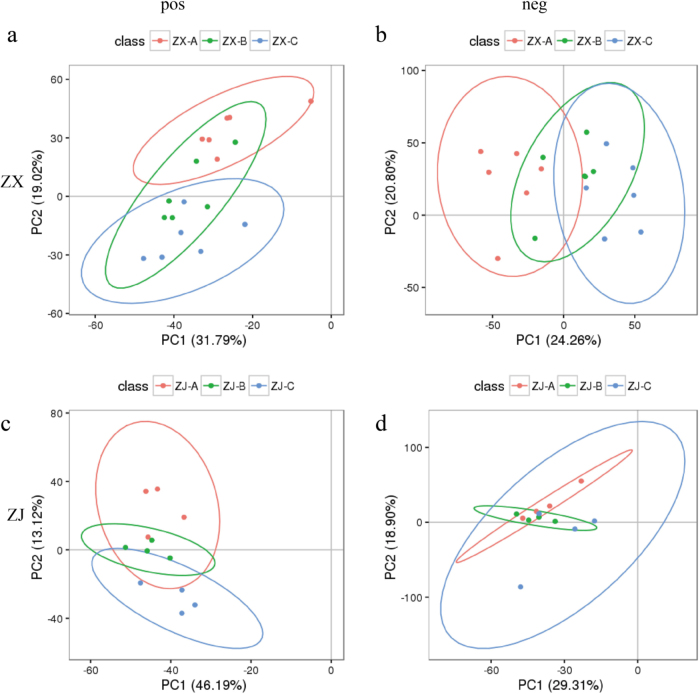
PCA score plots of leaves from tea cultivars “ZX” and “ZJ” in positive and negative ion modes. Principal component analysis (PCA) was applied to the LC–MS data set of “ZX” from all groups obtained using a C_18_ column in (**a**) positive and (**b**) negative ion modes. The PCA was applied to the LC–MS data set of “ZJ” from all groups obtained using a C_18_ column in (**c**) positive and (**d**) negative ion modes. Completely purple group (A, red circle), intermediately purple group (B, green circle), and completely green group (C, blue circle)

The PCA score plots exhibited an obvious separation between the completely purple group (A) and completely green group (C), whereas the intermediately purple group (B) overlapped with groups A and C in the two purple-leaf cultivars in both ion modes (Fig. 2). The first two components of the PCA score plot for cultivar “ZX” were 31.79 and 19.02%, respectively, in positive ion mode and 24.26 and 20.80% in negative ion mode (Fig. 2a and b). The first two components of the plot for “ZJ” explained 48.19 and 13.12%, respectively, of the variation in positive ion mode and 29.31% and 18.90% of the variation in negative mode (Fig. 2c and d). The high R^2^ and Q^2^ values (Supplementary Table 2) in both modes indicated the good quality and predictability of the multivariate model. These results demonstrated large metabolite differences between groups A and C but almost no significant differences between groups A and B or groups C and B.

### Metabolomic changes during purple tea cultivar growth

To elucidate the material basis of leaf color change in “ZX”, the metabolite changes in different colored leaves were studied. At the same time, the differences in metabolomic variations during leaf color change between “ZX” and “ZJ” were also investigated.

**Fig. 3:**
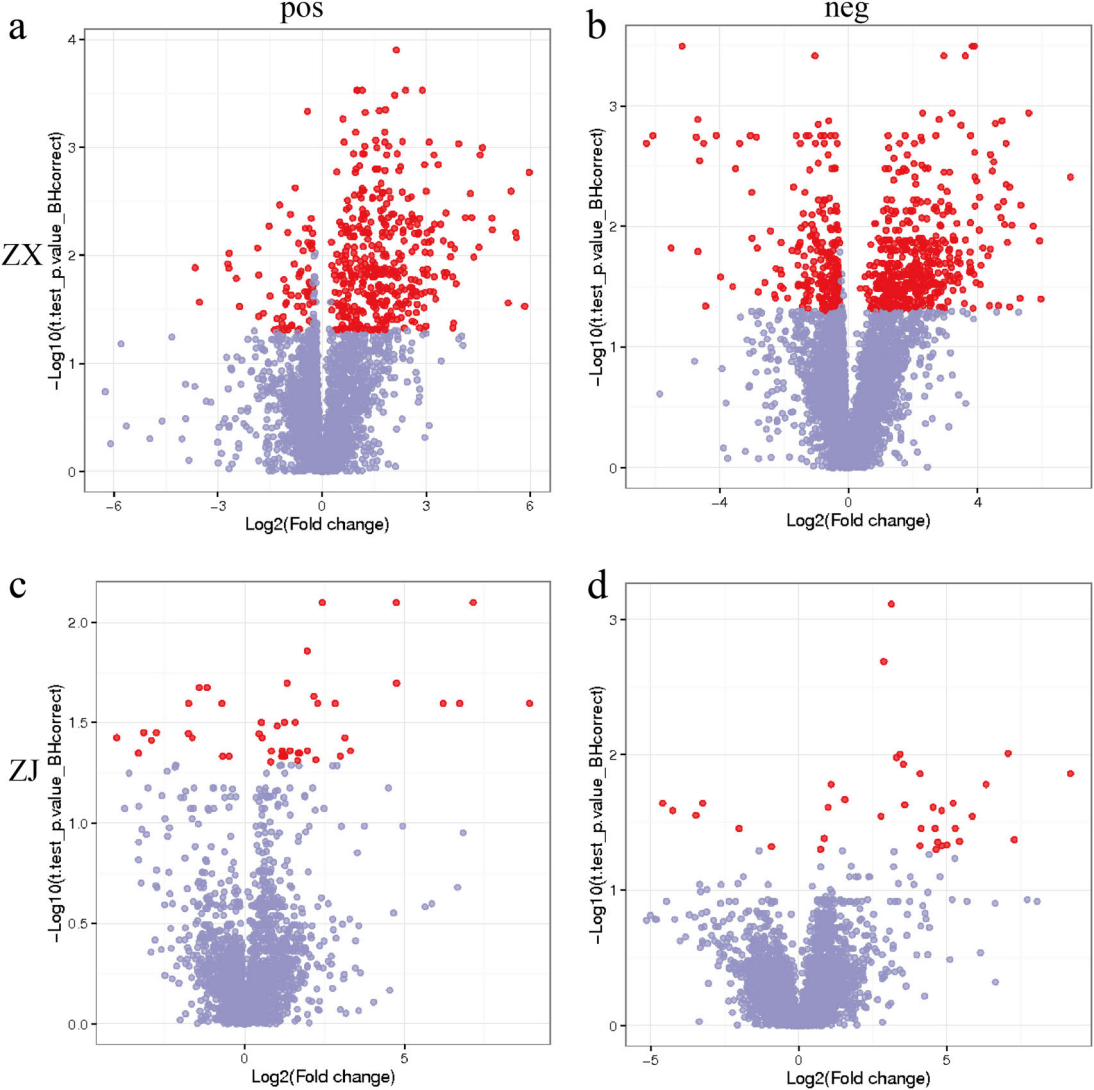
Metabolites that changed significantly during the leaf color change process. Differentially changed metabolites between purple and green leaves of cultivar “ZX” in the (**a**) positive and (**b**) negative ion modes. Differentially changed metabolites between completely purple and green leaves of cultivar “ZJ” in the (**c**) positive and (**d**) negative ion modes. Metabolites with q-value > 0.05 are indicated in gray; metabolites with fold change ≥ 1.2 or fold change ≤ 0.8 and q-value ≤ 0.05 are highlighted in red

Leaf metabolites passing the VIP > 1 threshold in the PLS-DA model and the *t*-test (*P* < 0.05) after FDR correction were considered as significantly different metabolites between two groups. There were 417 and 568 differential ions between purple (A) and green leaves (C) of “ZX” in positive and negative ion modes, respectively (Fig. 3a and b). However, no significantly different ions were found in groups A vs. B or groups C vs. B (Supplementary Figure 2). Only 45 and 35 differential ions were obtained between purple and green leaves of “ZJ” in positive and negative ion modes, respectively (Fig. 3c and d). The other contrast groups showed no significant differences in “ZJ”, similar to “ZX” (Supplementary Figure 2). A total of 32 and 27 differential ions in respective positive and negative ion modes were shared by the two cultivars (Supplementary Table 3). Based on the PCA model and the metabolic variations during the leaf color change, metabolite comparisons between purple and green leaves were further analyzed to determine the mechanism of leaf color change.

**Fig. 4:**
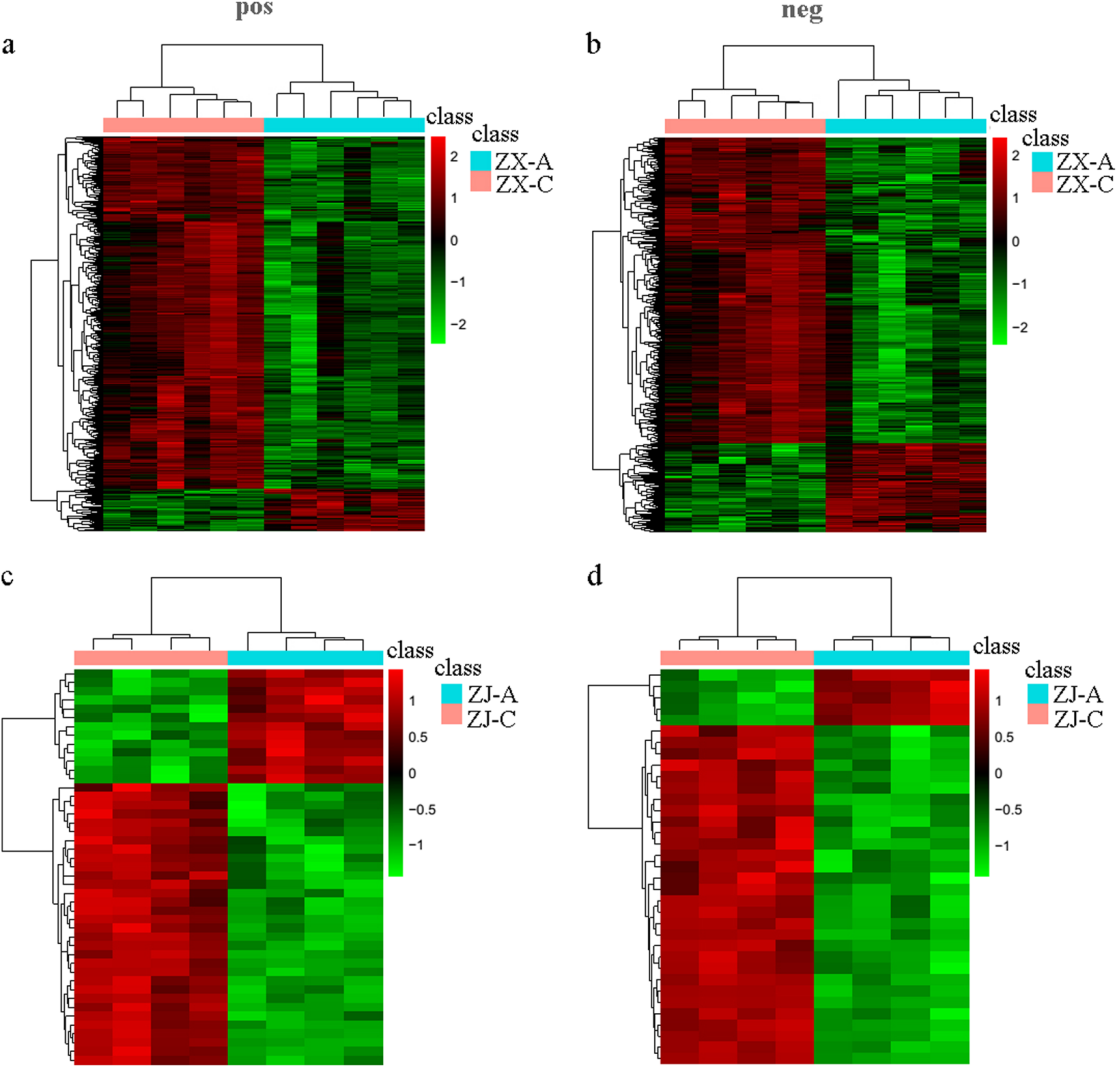
Hierarchical clustering of differential metabolites in different colored leaves. Differential metabolites between purple and green leaves of cultivar “ZX” in the (**a**) positive and (**b**) negative ion modes. Differential metabolites between purple and green leaves of cultivar “ZJ” in the (**c**) positive and (**d**) negative ion modes. The data were log 2 transformed, and similarity assessment for clustering was based on the Euclidean distance coefficient. Columns and rows represent individual metabolites and different samples, respectively

The selected differential metabolites were also analyzed by hierarchical clustering. There was a clear separation of metabolites between the purple and green leaves of the two cultivars in both ion modes (Fig. 4). Most of the differential metabolites increased during the leaf color change in both cultivars.

### Metabolites in the flavonoid biosynthetic pathway

**Fig. 5:**
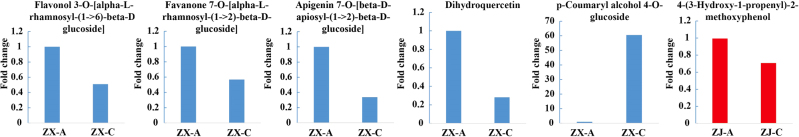
Fold changes of the flavonoids. Metabolites in the flavonoid biosynthetic pathway that changed significantly during the leaf color change from purple to green. The blue and red columns represent the metabolites that changed significantly in “ZX” and “ZJ”, respectively. Fold changes based on metabolite intensities in purple leaves were set as the control

The associations between flavonoids and leaf color phenotypes were investigated. The concentrations of flavonoids and flavonol glycosides changed significantly during the color shift from purple to green. Based on mass spectrometric data and database comparison, the following known flavonoids were assigned for cultivar “ZX”: apigenin 7-O-[beta-D-apiosyl-(1- > 2)-beta-D-glucoside] (F1), flavonol 3-O-[alpha-l-rhamnosyl-(1- > 6)-beta-D-glucoside] (F2), favanone 7-O-[alpha-L-rhamnosyl-(1- > 2)-beta-D-glucoside] (F3), dihydroquercetin (F4), and p-coumaryl alcohol 4-O-glucoside (F5) (Fig. 5). Flavonoids F1–F3 are intermediates of the flavone and flavonol biosynthetic pathways. F4 is involved in flavonoid biosynthesis, and F5 participates in phenylpropanoid biosynthesis. The levels of flavonoids F1–F4 were significantly higher in purple leaves, and the F5 level was higher in green than in purple leaves.

However, only one metabolite, 4-(3-hydroxy-1-propenyl)-2-methoxyphenol(F6), which is involved in phenylpropanoid biosynthesis, changed significantly during the leaf color change of “ZJ” and strongly accumulated in purple leaves. These results suggest that flavonoids contributed to the purple color of the leaves of both cultivars.

### Chlorophyll and carotenoid metabolism

**Fig. 6:**
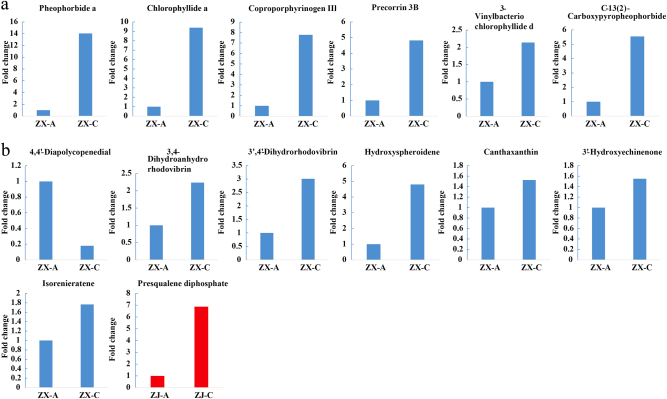
Fold changes of the chlorophyll- and carotenoid-related compounds. Metabolites in the (**a**) chlorophyll and (**b**) carotenoid metabolic pathways that changed significantly during the leaf color shift from purple to green. The blue and red columns represent the metabolites that changed significantly in “ZX” and “ZJ”, respectively. Fold changes based on metabolite intensities in purple leaves were set as the control

Obvious disparities of chlorophyll-related metabolites were observed between the two different colored leaves of cultivar “ZX” (Fig. 6a). Five intermediate metabolites of the porphyrin and chlorophyll metabolic pathways, including 3-vinylbacteriochlorophyllide d (C1), precorrin 3B (C2), C-13(2)-carboxypyropheophorbide a (C3), coproporphyrinogen III (C4), and pheophorbidea (C5), were detected in the negative ion mode, but only chlorophyllide a (Chlide a, C6) was detected in positive ion mode. In contrast to the flavonoids, all of these chlorophyll-related metabolites had higher levels in green compared to purple leaves. In particular, C5, C6, and C4 were 14.05-, 9.39-, and 7.78-fold higher, respectively, in green than in purple leaves (Fig. 6a).

The carotenoid-related metabolites also changed dramatically during the leaf color change of cultivar “ZX” (Fig. 6b). Seven compounds were characterized as significantly different metabolites: 4,4′-diapolycopenedial (C7), canthaxanthin (C8), 3′-hydroxyechinenone (C9), isorenieratene (C10), 3,4-dihydroanhydrorhodovibrin (C11), 3′,4′-dihydrorhodovibrin (C12), and hydroxyspheroidene (C13). The contents of each of these metabolites were higher in green than in purple leaves, except for C7. Thus, chlorophyll and carotenoid metabolisms were enhanced in green leaves of “ZX”.

However, no significant differences inmetabolites were observed in the porphyrin and chlorophyll metabolic pathways of cultivar “ZJ”. Only presqualene diphosphate (C14), involved in both carotenoid and steroid biosynthesis, showed a higher level in green than in purple leaves during the color change.

### Fatty acid metabolism

**Fig. 7:**
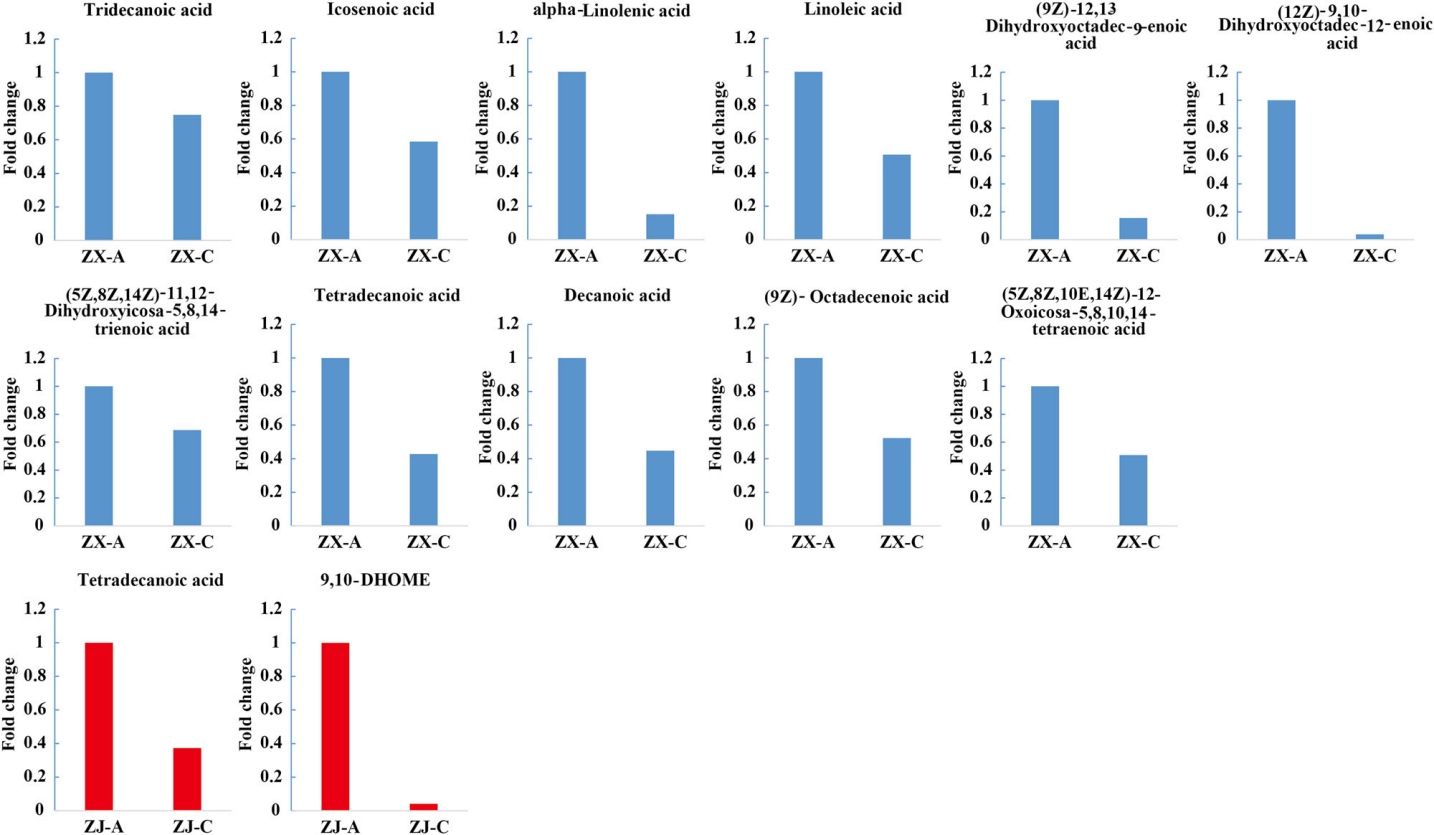
Fold changes of the fatty acids. Metabolites in the fatty acid metabolic pathway that changed significantly during the leaf color shift from purple to green. The blue and red columns represent the metabolites that changed significantly in “ZX” and “ZJ”, respectively. Fold changes based on metabolite intensities in purple leaves were set as the control

Eleven fatty acids showed significant discrepancies between the two colored leaves of cultivar “ZX” (Fig. 7): (12Z)-9,10-dihydroxyoctadec-12-enoic acid (FA1), linoleic acid (FA2), (9Z)-octadecenoic acid (FA3), icosenoic acid (FA4), alpha-linolenic acid (FA5), (9Z)-12,13-dihydroxyoctadec-9-enoic acid (FA6), tetradecanoic acid (FA7), decanoic acid (FA8), (5Z,8Z,10E,14Z)-12-oxoicosa-5,8,10,14-tetraenoic acid (FA9), (5Z,8Z,14Z)-11,12-dihydroxyeicosa-5,8,14-trienoic acid (FA10), and tridecanoic acid (FA11). Seven of these were detected in positive ion mode. According to the KEGG pathway analysis, four unsaturated fatty acids (FA1, FA2, FA5, and FA6) are involved in linoleic acid metabolism, and three saturated fatty acids (FA3, FA7, and FA8) are involved in fatty acid biosynthesis. FA4 participates in the biosynthesis of unsaturated fatty acids. FA9 and FA10 act as intermediates of arachidonic acid metabolism. All of these fatty acids significantly decreased during the leaf color change of “ZX” (Fig. 7).

Only two fatty acids showed significant changes in cultivar “ZJ”, i.e., FA7 and 9,10-DHOME (FA12), which showed higher levels in purple than in green leaves (Fig. 7). Thus, the contents of fatty acids were positively correlated with purple leaves and negatively correlated with green leaves.

### Steroid metabolism

**Fig. 8:**
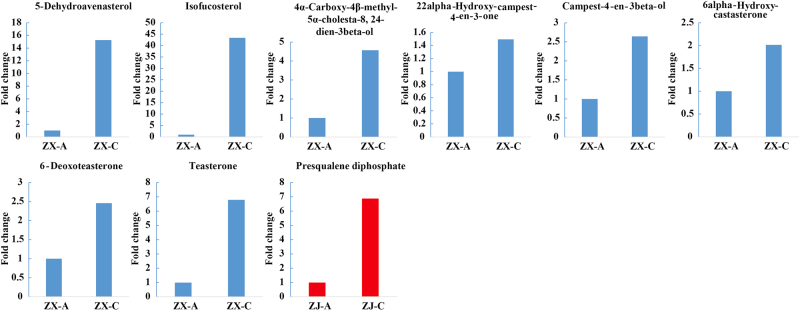
Fold changes of the steroids. Metabolites in the steroid metabolic pathway that changed significantly during the leaf color change from purple to green. The blue and red columns represent metabolites that changed significantly in “ZX” and “ZJ”, respectively. Fold changes based on metabolite intensities in purple leaves were set as the control

Steroids, which increase crop yield and act as plant-protection agents, are versatile hormone regulators.^36^ Intermediates involved in the steroid biosynthetic pathway, including 5-dehydroavenasterol (S1), isofucosterol (S2), and 4α-carboxy-4β-methyl-5α-cholesta-8,24-dien-3beta-ol (S3), exhibited higher levels in the green compared to the purple leaves of cultivar “ZX” (Fig. 8). The same variation models applied to compounds that are involved in the brassinosteroid biosynthetic pathway, including 22alpha-hydroxy-campest-4-en-3-one (S4), campest-4-en-3beta-ol (S5), 6alpha-hydroxy-castasterone (S6), 6-deoxoteasterone (S7), and teasterone (S8) (Fig. 8). These results indicate that steroid biosynthesis was enhanced in the green leaves of cultivar “ZX”. However, only one metabolite in “ZJ”, i.e., presqualene diphosphate (C14), which is involved in steroid biosynthesis, showed a similar change pattern to thesteroids in “ZX”. Thus, the steroid contents were negatively correlated with purple leaves and positively correlated with green leaves.

### Other metabolites that changed significantly during leaf color change

Since leaf color change is accompanied by numerous biochemical changes, many other metabolites also changed significantly in cultivar “ZX”, such as saponins, alcohols, and organic acids (Supplementary Table 4). Most of the saponins and saponin aglycones showed higher levels in green leaves. In contrast, gibberellin A12 aldehyde and 5α-acetoxy-10β,14β-dihydroxytaxadiene, which participate in diterpenoid biosynthesis, decreased in this process. Nerolidol, 24-hydroxy-beta-amyrin, and olean-12-ene-3beta, 24-diol, from the squiterpenoid and triterpenoid biosynthetic pathways, presented different change patterns during this process—nerolidol decreased and the remaining two increased.

### Analysis of pigment contents

To verify our hypothesis that the two purple cultivars have different mechanisms for leaf color change, the pigment contents in different colored leaves of “ZX” and “ZJ” were determined. The chlorophyll content increased significantly during the leaf color change of “ZX”; however, this increase was not significant in “ZJ”. During the leaf color change, the carotenoid contents decreased slightly but not significantly in either cultivar. Similarly, the anthocyanin content decreased in both cultivars during the color change process, but this decrease was only significant in “ZJ”.

### Relative expressions of genes related to pigment biosynthesis

**Fig. 9:**
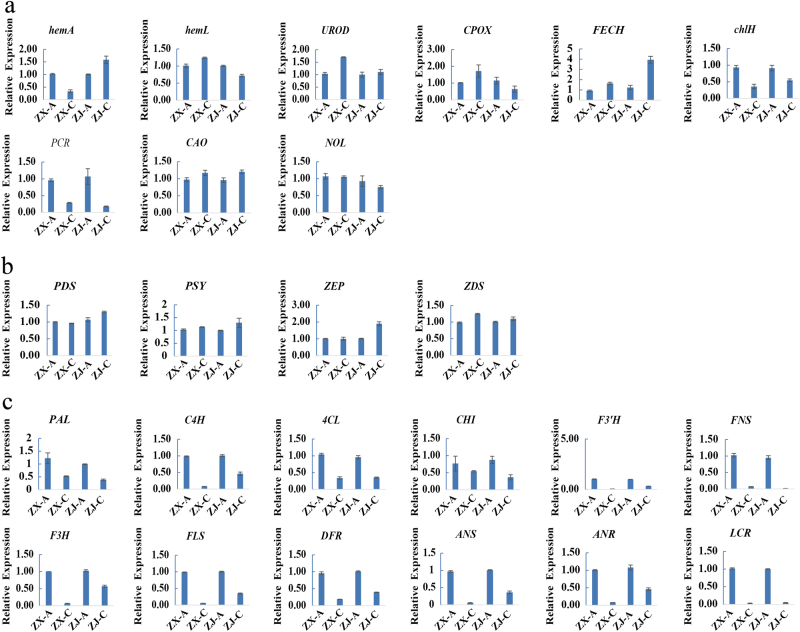
Relative expressions of genes related to pigment biosynthesis. Expression profiles of *hemA*, *hemL*, *UROD*, *CPOX*, *FECH*, *chlH*, *PCR*, *CAO*, *NOL*, *PDS*, *PSY*, *ZEP*, *ZDS*, *PAL*, *C4H*, *4CL*, *CHI*, *F3’H*, *FNS*, *F3H*, *FLS*, *DFR*, *ANS*, *ANR*, *LCR* genes involved in the (**a**) chlorophyll, (**b**) carotenoid, and (**c**) flavonoid biosynthetic pathways. All data are shown as the mean ± S.E.M. (*n* = 3)

To determine whether the expression of genes related to the biosynthesis of pigments was in accordance with metabolite accumulations, 25 genes were selected according to previous studies: 4 genes were involved in carotenoid biosynthesis, 9 in chlorophyll biosynthesis, and 12 in flavonoid biosynthesis.^2,37^ Their expressions were quantified. Three chlorophyll biosynthesis-related genes (*hemL*, *CPOX*, and *NOL*) were up-regulated in cultivar “ZX” but down-regulated in “ZJ” during leaf color change.* UROD*, *FECH*, and *CAO* were up-regulated, but *chlH* and *PCR* were down-regulatedin both cultivars during this process (Fig. 9a). Interestingly, expressions of the carotenoid biosynthesis-related genes were slightly up-regulated (Fig. 9b), and genes involved in flavonoid/anthocyanin biosynthesis were down-regulated (Fig. 9c) in both cultivars during the color change process.

## Discussion

**Fig. 10:**
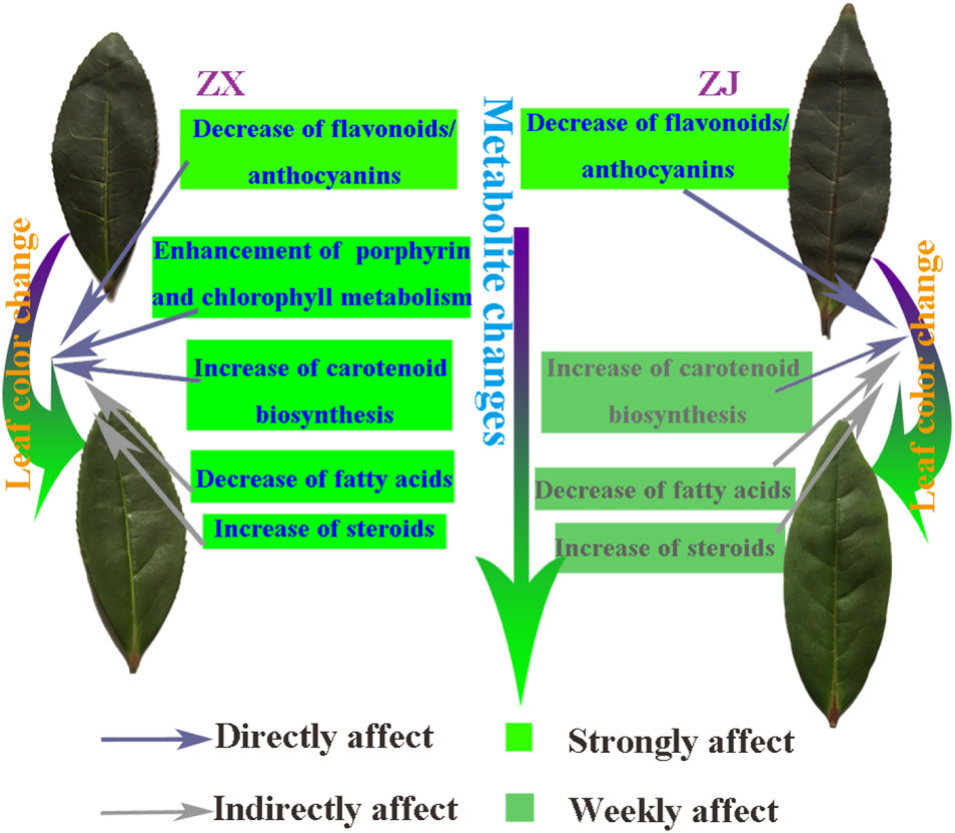
The comparison of leaf color change mechanisms. Models of possible mechanisms of leaf color change for tea cultivars “ZX” and “ZJ”. The decrease of flavonoids/anthocyanins strongly and directly caused the color change in “ZJ”. The slight strengthening of carotenoid biosynthesis, decreases in fatty acids, and weak increases in steroids caused the color change in “ZJ”. However, in “ZX”, the decrease of flavonoids/anthocyanins and enhanced porphyrin and chlorophyll metabolism strongly and directly caused the color change. This was also strongly promoted by the strengthening of carotenoid biosynthesis, decreases in fatty acids, and increases in steroids during the leaf color change from purple to green

The variations of leaf color in tea plants have attracted extensive attention due to their specific biochemical compounds that greatly influence tea quality. In the present study, the mechanism of leaf color, changing from purple to green, at the metabolome level of “ZX”, a new purple cultivar, was elucidated, and the biochemical changes that occurred during the process were also investigated. In addition, differences in the color change mechanisms between cultivars “ZX” and “ZJ” were compared (Fig. 10).

### The purple-leaf phenotype is closely related to flavonoid metabolism

The development of purple leaves is generally related to pigment metabolism. The color of plant tissues can be attributed to three major pigments: chlorophylls, carotenoids, and flavonoids.^38,39^ Flavonoids/anthocyanins are generally responsible for the red, blue, and purple pigments in leaves^40^—when their concentrations are sufficiently high to mask the green color of chlorophylls, leaf coloration then depends mainly on flavonoids/anthocyanins.^41^ In this study, several flavonoids showed significantly higher levels in purple than in green leaves in both cultivars. Therefore, flavonoids might overlap with the chlorophyll coloration and lead to the formation of purple color in leaves.

Anthocyanins are the ultimate products of the flavonoid biosynthetic pathway, which is downstream of the phenylpropanoid pathway.^42,43^ The low level of p-coumaryl alcohol 4-O-glucoside, which is used in the biosynthesis of downstream flavonoids, and higher levels of flavonoids could provide more substrates and lay the foundation for anthocyanin accumulation in purple leaves. Consistently, the purple leaves contained higher anthocyanin contents than the green leaves. In a previous study, Li et al.^6^ reported that the contents of anthocyanins and total flavonoids increased by 409.62% and 36.47%, respectively, in purple compared with green leaves of cultivar “ZJ”. Moreover, anthocyanin-rich leaves of *Eucalyptus* saplings^44^ and eggplants^45^ were reported to contain high concentrations of other phenolic compounds, indicating that anthocyanins shared common starting steps with other phenolics in the phenylpropanoid biosynthetic pathway.^41^ These studies demonstrate that anthocyanins and flavonoids likely co-exist and both are present at high levels in purple leaves. The relative expressions of genes in the flavonoid and anthocyanin biosynthetic pathways were up-regulated in purple leaves of both “ZX” and “ZJ” (Fig. 9c), verifying that flavonoids accompanied by anthocyanin accumulated in the purple leaves of both cultivars. The change in leaf color to green might be caused by low levels of flavonoid/anthocyanin biosynthesis, leading to low flavonoid/anthocyanin accumulation in the green leaves of the two purple-leaf tea cultivars.

Flavonoids/anthocyanins play critical roles in resisting environmental stresses, such as chilling, drought, and UV-B irradiation, and also regulate plant growth.^46,47^ The higher contents of flavonoids/anthocyanins in the young leaves of the two purple cultivars could protect these leaves from high-light damage and other adverse environmental stresses. The health-related functions of flavonoids/anthocyanins can promote tea quality.^48–50^ These two purple cultivars are valuable materials for tea plant breeding and germplasm construction.

### The green leaf phenotypeis closely related to active chlorophyll and carotenoid metabolism in “ZX”

The change in the leaf color of tea plants from purple to green might also be due to changes in different pigment ratios. Leaves appear green mainly due to high concentrations of chlorophylls. Tea leaves present an albino or chlorina phenotype due mainly to altered chlorophyll metabolism.^2,4^ In this study, the altered chlorophyll metabolism also contributed to the leaf color change from purple to green in “ZX”, during which the metabolites mapped to the porphyrin and chlorophyll metabolic pathways significantly increased. In addition, the chlorophyll content was significantly higher in green than in purple leaves of “ZX”. Genes involved in the chlorophyll biosynthetic pathway, such as *hemL*, *CPOX*, *UROD*, *FECH*, *CAO*, and *NOL*, were up-regulated during the color change. However, the key gene *ChlH* was down-regulated. The result is consistent with findings that *ChlH* was up-regulated in pale white compared to green shoots in cultivars “ZH2” and “AnjiBaicha”^2,3^ because chlorophyll synthesis depends on active magnesium-chelatase requiring a balanced proportion of each subunit, including ChlH.^51^ The higher level of *ChlH* might upset the balance of the three subunits and ultimately decrease biosynthesis of chlorophyll in purple leaves. Thus, we attributed the leaf color change of “ZX” to the strengthening of chlorophyll metabolism.

However, no differential metabolites related to the porphyrin and chlorophyll metabolic pathways were identified in “ZJ”. In addition, the chlorophyll content did not increase significantly during the leaf color change, and the genes involved in chlorophyll biosynthesis were down-regulated in “ZJ” (Fig. 9a). These results indicated that the slight increase in the chlorophyll content did not play a critical role in the leaf color change of “ZJ”.

Chlorophyll plays a pivotal role in converting light energy to stored chemical energy,^9^ and carotenoids function as accessory light-harvesting pigments by trapping light energy and transferring it to chlorophylls. Carotenoids, which are principally synthesized in the chloroplast membrane, are a group of important isoprenoids involved in photosynthesis.^52,53^ In the present study, metabolites in the carotenoid biosynthetic pathway increased significantly, and this was verified by qRT-PCR. Four genes involved in carotenoid biosynthesis detected in this study were slightly up-regulated in the green leaves of both cultivars. However, the carotenoid content decreased slightly during the leaf color change in both cultivars. Carotenoids may act as a supplement to the declining flavonoid/anthocyanin protection system and may be consumed subsequently in the leaf color change process because carotenoids in green leaves help ensure efficient photosynthesis, scavenge various reactive oxygen species,^54^ and protect chlorophylls from photooxidation.^55^ In addition, carotenoids can also function as precursors for apocarotenoids such as the plant hormone abscisic acid,^54,56^ which is essential for plant growth, development, and antioxidant capacity.^57–59^ In short, enhanced carotenoid biosynthesis contributed to maintaining the chlorophyll level, which resulted in the green leaf coloration.

### Changes in fatty acids and steroids might contribute to chlorophyll biosynthesis

Fatty acids play fundamental roles, such as providing energy or storage during plant development.^60^ For example, long-chain fatty acids are usually involved informing cell membrane phospholipids and regulating a number of cellular functions.^61^ They are speculated to be related to chlorophyll biosynthesis. In this study, an interesting change pattern was found for fatty acids during the leaf color change process in cultivar “ZX”. Fattyacids, including linoleic, alpha-linolenic, and (9Z)-octadecenoic acids,all decreased. This is similar tothe discovery that genes related to “fatty acid degradation” were up-regulated during the leaf color change from yellow to green in the cultivar “Baijiguan”.^5^ These results indicate that fatty acid degradation might play a role in the green leaf phenotype.The function of fatty acids in the green color formation differed between the two cultivars, e.g., fatty acid degradation modified the membrane fluidity and then repaired the chloroplast structure of “Baijiguan”. However, the phenomenon in our study may have occurred because fatty acids were converted to acetyl-CoAs or acyl CoAs, as previously reported.^60^ Acetyl-CoA is a central key biomolecule, playing critical roles in catabolic and anabolic pathways of carbohydrates, lipids, and amino acids in plants,^62,63^ which can provide sufficient substrates for chlorophyll biosynthesis.^64^ Consistently, chlorophyll biosynthesis was active, and the final chlorophyll content was significantly higher in green than in purple leaves of “ZX”. Other degradation substrates of fatty acids, i.e., acyl CoAs, used in steroid biosynthesis, were reported previously.^65^ Scaller found that steroids, the natural substances essential for plant growth and development, played a role in regulating metabolic processes, such as respiration.^66^ Coincidently, in the present study, we also found that the levels of intermediates of the steroid and brassinosteroid biosynthetic pathways increased during the leaf color change of “ZX”.

These results verified our speculation that fatty acids degraded to supply substrates for metabolism of other compounds—they might contribute to or regulate chlorophyll biosynthesis. Furthermore, the extent of changes in fatty acids and steroids in “ZJ” were not as great as in “ZX”, which indicates that the mechanisms of leaf color change differ between the two cultivars.

## Conclusions

Comparative metabolomic analyses of purple and green leaves of two purple-leaf cultivars were performed, and various metabolites and pathways potentially responsible for leaf color change were identified. Metabolite changes during the leaf color change indicated different underlying mechanisms between the two cultivars. In “ZJ”, the decrease of flavonoids/anthocyanins mainly led to the leaf color change. However, the decrease of flavonoids/anthocyanins, the strengthening of porphyrin and chlorophyll metabolism, greater carotenoid biosynthesis, decreases in fatty acids, and increases in steroids strongly promoted the leaf color change from purple to green in “ZX”. This study may facilitate future research on the regulatory mechanisms of color change in purple-leaf cultivars.

## Electronic supplementary material

Supplementary Information

Supplementary Figure 1

Supplementary Figure 2

Supplementary Table 1

Supplementary Table 2

Supplementary Table 3

Supplementary Table 4
