# Supplementary material for: Metabolic analyses reveal different mechanisms of leaf color change in two purple-leaf tea plant (Camellia sinensis L.) cultivars
Source: Hortic Res. 2018 Feb 7;5:7. doi: 10.1038/s41438-017-0010-1 (PMC5802758; doi:10.1038/s41438-017-0010-1)
Supplement: Supplementary file 1 — Supplementary Information [file 41438_2017_10_MOESM1_ESM.docx]

**Supplementary Figure 1**LC-MS chromatograms of QC samples in both positive and negative ion modes.

**Supplementary Figure 2**No significantly different ions were found in groupsA vs. B or groups C vs. B in both cultivars ‘ZX’ and ‘ZJ’ in the positive and negative ion modes. Metabolites with q-value > 0.05 are indicated in gray.
