# Supplementary figures and images for: Metabolic analyses reveal different mechanisms of leaf color change in two purple-leaf tea plant (Camellia sinensis L.) cultivars
Source: Hortic Res. 2018 Feb 7;5:7. doi: 10.1038/s41438-017-0010-1 (PMC5802758; doi:10.1038/s41438-017-0010-1)

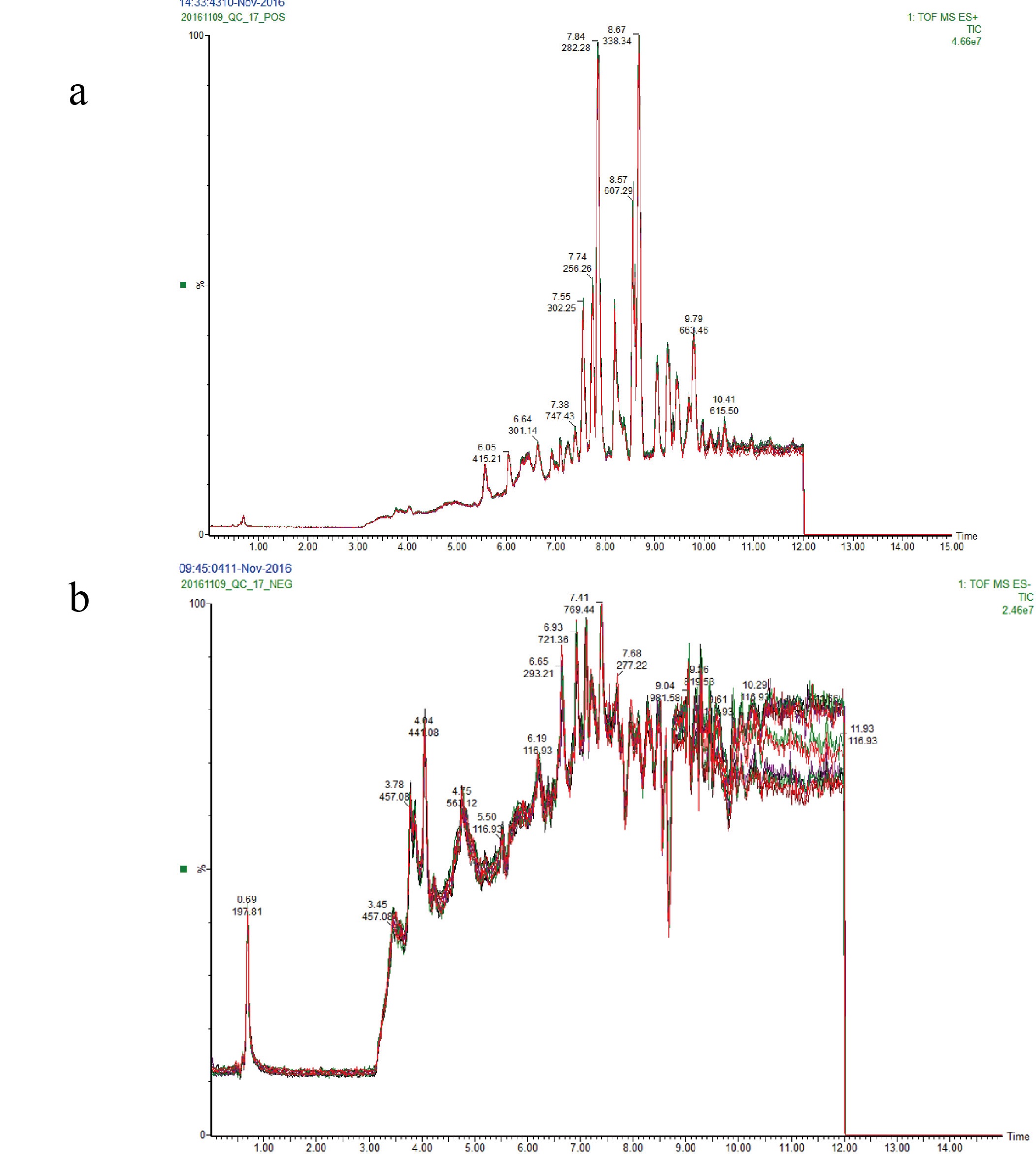

Supplement: Supplementary file 2 — Supplementary Figure 1 [file 41438_2017_10_MOESM2_ESM.jpg]

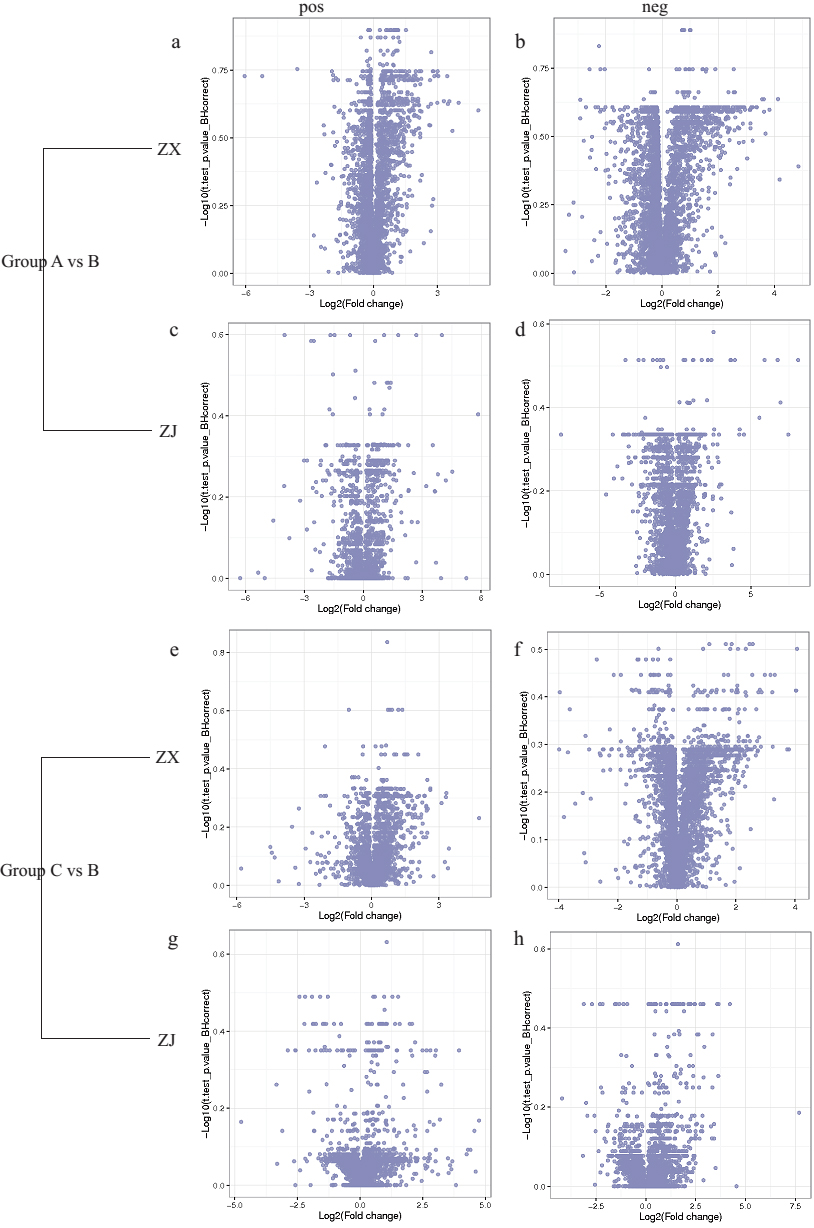

Supplement: Supplementary file 3 — Supplementary Figure 2 [file 41438_2017_10_MOESM3_ESM.jpg]
