# Supplementary material for: Metabolic analyses reveal different mechanisms of leaf color change in two purple-leaf tea plant (Camellia sinensis L.) cultivars
Source: Hortic Res. 2018 Feb 7;5:7. doi: 10.1038/s41438-017-0010-1 (PMC5802758; doi:10.1038/s41438-017-0010-1)
Supplement: Supplementary file 4 — Supplementary Table 1 [file 41438_2017_10_MOESM4_ESM.docx]

**Supplementary Table 1 Primer sequences for quantitative RT-PCR.**

| **Genes** | **Forward Primer (5’ - 3’)** | **Reverse Primer (5’- 3’)** |
| --- | --- | --- |
| **GAPDH** | TTGGCATCGTTGAGGGTCT | CAGTGGGAACACGGAAAGC |
| **β-actin** | GCCATCTTTGATTGGAATGG | GGTGCCACAACCTTGATCTT |
| **hemA** | CATTTCAACAGGTGTAGTGTGG | CGCTCGAACAACTCAAGGCATC |
| **hemL** | GGTTATATAAGCGGGATGTTTG | CAGTGTCACTCTTCTTCGCATC |
| **UROD** | GAGGAGTCAGTTCCGTATGTTG | AACAAACCCAGTACGGCCGCTT |
| **CPOX** | TTGGACTCAAGACGGGAGGTAG | GTTTCCATTCTTCGCTTCCCTC |
| **FECH** | AGTTCTTCAACGCTTCCAAGAG | CCACTTTCATTTCAGATTTGGC |
| **chlH** | GGGATGTTTGGGTGAATAGTCC | TTCCTTCCCAAGAAAACCAACC |
| **PCR** | TGACTTGCTCTCTTTGGGGATG | ATCCATGGCTCTCCAGTCTGCT |
| **CAO** | ACGTGTCTGGTCCTGTTCAACC | GTGTCATCCTTCAAGTCCGTAG |
| **NOL** | TTTCACGGATGCCATGGAGATG | ATTATGAAGGCACAAACGACAG |
| **PSY** | GTTCTTCCGGGGACTTCGAG | GACGCATTAGGCCCATCAAC |
| **PDS** | AGGTGGACAGGCCTATGTTG | TGGCTGGCAAAGTCTCTCTG |
| **ZDS** | CAGATACAACGGCTGGGTCA | CAGGTGTAAGCACGCATTGG |
| **ZEP** | TGCAGGAAAGATGCAGTGGT | GTTAAAAGTTGGTGCGCGGT |
| **PAL** | CACTCTGCCAAGCCGTAGAT | TGCACTGCAAGGGTCATCAA |
| **C4H** | GAGCATGGACAACAATAGTCTG | ACGTTGTCTTCATTGATCTCTC |
| **4CL** | TACAAAGGGTTCCAAGTAGCTC | AACTTCTCCTGCAGCCTCATCT |
| **CHI** | CAGAATTGTTGAAGGAGAAACC | TCAGCAACCTAACCACTAGTGT |
| **F3’H** | GCAATGATTTCGAGCTCATACC | CTAGTGTGCCCAAAATGTACTC |
| **FNS** | ACTTCTTGCCTGAGCGATTCTTA | GAAACACTGAATCATTGCCGCT |
| **F3H** | CTGGAAACGATGAATCCACCTT | GACTCGCTCGTGAGTTTTTTGC |
| **FLS** | GTCATGCACCCTCCTAAGAAG | CTGTCACTCCCCTGTATGAAG |
| **DFR** | TCCCATGATGCTACCATCCATG | TCAGTGGGGACATTGTACTCAG |
| **ANS** | ACGAGGGCAAATGGGTCA | TCCTTGGGTGGTTCGCAGA |
| **ANR** | CCATACCCTCCAGTGCTACGAG | TTGTGAAGATGTTGCTAGAGAG |
| **LCR** | CAGAATCATGTTGTTGAAGTAC | AGATGGATTAGATATGGTTCAC |
