# Supplementary material for: Metabolic analyses reveal different mechanisms of leaf color change in two purple-leaf tea plant (Camellia sinensis L.) cultivars
Source: Hortic Res. 2018 Feb 7;5:7. doi: 10.1038/s41438-017-0010-1 (PMC5802758; doi:10.1038/s41438-017-0010-1)
Supplement: Supplementary file 5 — Supplementary Table 2 [file 41438_2017_10_MOESM5_ESM.docx]

| mode | group | R^2^ | Q^2^ |
| --- | --- | --- | --- |
| pos | ZJ-C_ZJ-A | 0.997 | 0.680 |
| pos | ZX-C_ZX-A | 0.991 | 0.788 |
| neg | ZJ-C_ZJ-A | 0.968 | 0.721 |
| neg | ZX-C_ZX-A | 0.983 | 0.845 |

**Supplementary Table 2 The R^2^ and Q^2^ values for the PCA model of two cultivars in positive and negative ion modes.**
